# A Cooperative Model of GP-Led Memory Clinic and University Psychogeriatric Unit: Expanding Services to Rural Greece

**DOI:** 10.1192/j.eurpsy.2026.10669

**Published:** 2026-07-31

**Authors:** E. Konidari, S. Vasileios, K. Savvopoulou, P. Felemegkas, A. Kostakiotis, P. Alexopoulos

**Affiliations:** 1 Mental Health Services, University Hospital of Patras, School of Health Sciences, University of Patras, Patras; 2 Primary Healthcare Center of Erymanthia, Primary Healthcare Center of Erymanthia, Erimanthia, Greece; 3 Trinity College Dublin, The University of Dublin, Global Brain Health Institute, School of Medicine, Dublin, Ireland; 4 Technical University of Munich, Department of Psychiatry and Psychotherapy, Klinikum rechts der Isar, Munich, Germany

## Abstract

**Introduction:**

Primary healthcare plays a pivotal role in dementia management, particularly in low-resource settings, however specialized cognitive services are usually confined to tertiary hospitals, limiting access for older adults. The INTegRated InterveNtion of pSychogerIatric Care (INTRINSIC) program, launched in 2022, supports the development of telehealth-based, primary care–embedded services across Greece. Within this framework, the Primary Healthcare Center of Erymanthia established a general practitioner (GP)-led memory clinic in close cooperation with the Psychogeriatric Unit of the University Hospital of Patras.

**Objectives:**

To report the initial outcomes of expanding the Erymanthia memory clinic into a regional model through cooperative outreach with the university hospital and to assess its potential for wider implementation.

**Methods:**

From June to September 2025, joint teams from Erymanthia and the University Hospital of Patras conducted outreach visits to four rural healthcare centers (Andritsaina, Krestena, Simopoulo, and Ano Xora). Older adults referred for memory concerns underwent comprehensive neurocognitive and psychiatric evaluation. Individualized management plans, including pharmacological and psychosocial interventions, were developed collaboratively and shared with local primary care teams, who participated in assessment, decision-making, and follow-up care.

**Results:**

Forty-four older adults were assessed, several new diagnoses were established, including dementia, mild cognitive impairment, and mood disorders. Pharmacological treatments such as cholinesterase inhibitors, antidepressants were initiated, while referrals for neuroimaging and laboratory testing addressed previously unmet diagnostic needs. Engagement of local primary healthcare professionals reinforced continuity of care and enhanced their skills in psychogeriatric management. The cooperative outreach model proved feasible, acceptable, and adaptable to diverse primary care settings.

**Conclusions:**

The Erymanthia memory clinic has evolved into a network linking primary and university-based services, representing the first cooperative model of its kind in Greece. By combining GP-led services, psychogeriatric expertise, and telehealth support, it addresses structural gaps in psychogeriatric care and builds capacity in underserved areas. Its innovative and scalable design makes it highly suitable for extension to other rural and insular regions, potentially offering a transferable model for improving equity of access to mental and cognitive healthcare in other European contexts.

**Disclosure of Interest:**

None Declared

